# A Significant Decline in the Number of Newly Dispensed Analgesics During the First COVID‐19 Lockdown in The Netherlands

**DOI:** 10.1002/ejp.70139

**Published:** 2025-09-30

**Authors:** Maureen N. Zijlstra, Pantea Kiani, Pauline A. Hendriksen, Dana M. Dijkgraaf, Johan Garssen, Joris C. Verster

**Affiliations:** ^1^ Division of Pharmacology, Utrecht Institute for Pharmaceutical Sciences Utrecht University Utrecht the Netherlands; ^2^ Danone Global Research and Innovation Center Utrecht the Netherlands; ^3^ Cognitive Neurophysiology, Department of Child and Adolescent Psychiatry, Faculty of Medicine TU Dresden Dresden Germany; ^4^ Centre for Mental Health and Brain Sciences Swinburne University Melbourne Victoria Australia

**Keywords:** analgesics, COVID‐19, delayed healthcare, lockdown, Netherlands, pain, pharmacy dispensing, prescriptions

## Abstract

**Background:**

The COVID‐19 pandemic disrupted healthcare systems worldwide, including the postponement of non‐urgent care and reallocation of resources toward COVID‐19 patients. The aim of this study was to assess the impact of the first Dutch COVID‐19 lockdown on the initiation of new analgesic prescriptions.

**Methods:**

This study analysed dispensing data from 1890 Dutch pharmacies, covering approximately 96% of the population (5.46 million patients). The number of first‐time prescription analgesics dispensed (ATC2 N02 class, e.g., opioids, anilides) was compared between the first halves of 2019 and 2020. First‐time users were defined as patients who had not received the drug in the preceding year. Data were stratified by age group (children, adolescents, adults, elderly), sex and time periods: weeks 1–11 (pre‐lockdown 2020), 12–19 (lockdown) and 20–26 (post‐lockdown).

**Results:**

The total number of first‐time dispensed analgesic drugs was significantly lower in 2020 (367,094) than in 2019 (388,973, *p* = 0.021), with a notable reduction occurring during the lockdown period (*p* = 0.003). Significant declines in first‐time dispensed analgesic drugs were observed among adolescents and adults during lockdown (*p* < 0.001), particularly among females. In contrast, no significant changes were found among children and the elderly. The number of first‐time dispensed analgesic drugs during the pre‐ and post‐lockdown periods was comparable between the 2 years.

**Conclusion:**

Delayed healthcare during the lockdown was associated with a decrease in first‐time dispensed analgesic drugs, especially among adolescents and adults. This may indicate untreated pain or increased reliance on over‐the‐counter alternatives during this period.

**Significance Statement:**

This Nationwide Dutch study showed a significant decline in first‐time dispensed analgesic drugs during the first lockdown period of the COVID‐19 pandemic, especially among adolescents and adults. This may indicate untreated pain due to delayed healthcare or an increased reliance on over‐the‐counter alternatives during this period.

## 1 Introduction

The 2019 coronavirus disease (COVID‐19) pandemic placed unprecedented demands on healthcare systems worldwide. In response, many countries implemented lockdown measures to curb the spread of the SARS‐CoV‐2 virus. In the Netherlands, the first lockdown lasted from week 12 to 19 of 2020 (Rijksinstituut voor Volksgezondheid en Milieu [RIVM] 2020). During this period, businesses were closed, people were advised to work from home, and (large) social gatherings were prohibited (RIVM 2020). However, essential businesses such as supermarkets, physician offices, hospitals and pharmacies remained open (RIVM 2020). As healthcare providers prioritized the treatment of COVID‐19 patients, non‐urgent healthcare services were postponed, resulting in an 11% decrease in general practitioner visits (RIVM 2021). This led to delays in care for non‐COVID patients.

Several aspects of Dutch healthcare were disrupted. For example, surgical care decreased by 13.6%, and 9.6% of planned surgeries were delayed compared to the 2 years prior (De Graaff et al. 2022). Additionally, a decrease in cancer diagnoses was reported during the first lockdown, largely attributed to the suspension of national screening programs aimed at alleviating pressure on the healthcare system (Dinmohamed et al. 2020). A survey on the self‐reported healthcare delays during the pandemic found that 31% of respondents in the Netherlands experienced postponed healthcare (Visscher et al. 2023). This delay was attributed both to healthcare providers (14%) and to patients themselves (12%) who chose to avoid medical settings (Visscher et al. 2023). Another study reported that 20.2% of people avoided seeking medical care during the pandemic, often even for urgent health concerns (Splinter et al. 2021). These findings suggest a need to explore whether individuals experiencing pain refrained from seeking medical treatment during the initial lockdown in the Netherlands.

Pain is a common and central symptom in many diseases and injuries, and delayed healthcare is likely to exacerbate the pain experienced by patients. This was observed in a study conducted in Germany, which found that patients with cancelled or postponed treatments reported significantly increased pain and symptom deterioration (Kleinmann et al. 2021). Similarly, research on postsurgical, musculoskeletal and neuropathic pain during the COVID‐19 pandemic revealed a worsening of pain symptoms, with patients experiencing greater pain (Chatkoff et al. 2022). Additionally, most elective interventional surgeries were postponed or cancelled during the pandemic. A study investigating the rescheduling of these surgeries found that most patients preferred to be rescheduled, with only 3% reporting spontaneous improvement of their symptoms (Gitkind et al. 2023). This suggests that the delay in elective surgeries led to prolonged pain, as the COVID‐19 restrictions prevented timely medical intervention. These findings indicate that delayed healthcare likely contributed to patients continuing to manage the pain without appropriate treatment.

Taken together, it can be inferred that avoidance of healthcare during the first Dutch COVID‐19 lockdown contributed to a reduction in the number of patients receiving analgesic medications, while delayed healthcare further exacerbated the decrease in analgesic prescriptions. To further evaluate this, dispensing data from Dutch pharmacies were analyzed in the first half of 2019 with those in the first half of 2020.

## 2 Methods

Data were collected on the number of patients who received prescription analgesics for the first time (i.e., newly dispensed analgesic drugs by Dutch pharmacies) during the first half of 2019 and the first half of 2020. A first‐time user was defined as a person who had not received the drug for at least 1 year prior to the prescription date. Dispensing data were obtained from the Dutch Foundation for Pharmaceutical Statistics (Stichting Farmaceutische Kentallen [SFK]), encompassing data from 1890 pharmacies and covering 98% of Dutch public pharmacies (SFK 2023).

Patients included in the analyses received one or more first‐time dispensed analgesics classified under ATC2 N02, according to the Anatomical Therapeutic Chemical (ATC) Classification System of the World Health Organization (WHO 2025a, 2025b). Common analgesics in category N02 include paracetamol and opioids such as morphine, oxycodone, codeine and tramadol. The category also includes other analgesics and antipyretics (e.g., salicylic acid and derivatives, pyrazolones, anilides and gabapentinoids), as well as antimigraine preparations. These comprise the most commonly prescribed analgesics worldwide and in the Netherlands for acute and chronic pain. In the Netherlands, frequently prescribed analgesics include non‐steroid anti‐inflammatory drugs (NSAIDs) such as diclofenac and ibuprofen (45.7%) and opioid drugs such as morphine and oxycodone (45.8%) (Veldkamp et al. 2024). Over‐the‐counter (OTC) analgesics were not included, as they are not captured in the SFK‐database.

For all patients, age (in years), sex (male or female) and the week of the year in which they received their first‐time dispensed analgesic were recorded. Prescription data were collected for the first half of 2019 (serving as baseline reference data) and for the first half of 2020, which included the period of the first Dutch COVID‐19 lockdown.

According to the WHO age classification (WHO 2025c, 2025d), patients were categorised into the following age groups to explore age‐specific patterns: children (0–9 years), adolescents (10–19 years), adults (20–64 years) and elderly (≥ 65 years). To assess the impact of the first Dutch COVID‐19 lockdown on the number of new patients initiating analgesic use, data were compared across three distinct time periods: (1) weeks 1–11, representing the pre‐lockdown phase in 2020; (2) weeks 12–19, corresponding to the lockdown period and (3) weeks 20–26, representing the post‐lockdown phase.

For each year (2019 and 2020), comparisons between the three time periods were performed using a GLM repeated measures analysis, applying Bonferroni correction for multiple comparisons (two‐sided, with statistical significance set at *p* < 0.025). In addition, for each time period, the weekly number of patients receiving first‐time dispensed analgesics in 2019 and 2020 was compared using paired *t*‐tests. A *p*‐value < 0.05 (two‐sided) was considered statistically significant. All analyses were stratified by age group, and separate analyses were conducted for males and females.

## 3 Results

**FIGURE 1:**
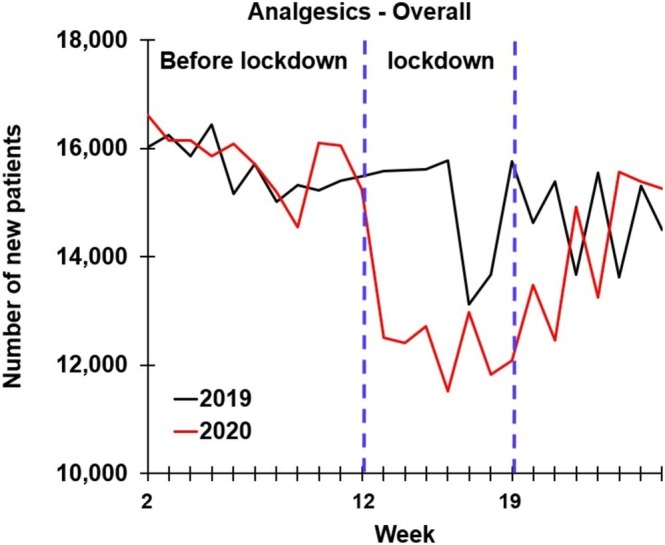
Number of newly dispensed analgesics in 2019 and 2020.

Data from 5.46 million people living in the Netherlands who received prescription drugs in 2019 and/or 2020 was included in the analyses. A summary of the findings is presented in Table 1 and Figure 1.

**TABLE 1:** Number of first‐time dispensed analgesic drugs in the Netherlands.

Age group	Population	Year	Week 1–11	Week 12–19	Week 20–26
Children	Overall	2019	291	239	200
		2020	271	182	181
		*p*	0.428	0.097	0.109
		% change	−6.9%	−23.8%	−9.5%
	Male	2019	166	147	124
		2020	155	105	113
		*p*	0.617	0.089	0.297
		% change	−6.6%	−28.6%	−8.9%
	Female	2019	125	92	76
		2020	116	77	68
		*p*	0.502	0.260	0.084
		% change	−7.2%	−16.3%	−10.6%
Adolescents	Overall	2019	4293	3121	2896
		2020	4008	1493	2255
		*p*	0.014*	< 0.001*	0.018*
		% change	−6.6%	−52.2%	−22.1%
	Male	2019	1778	1317	1220
		2020	1678	658	904
		*p*	0.165	< 0.001*	0.013*
		% change	−5.6%	−50.0%	−26.0%
	Female	2019	2515	1804	1676
		2020	2230	835	1351
		*p*	0.020*	< 0.001*	0.033
		% change	−11.3%	−53.7%	−19.4%
Adults	Overall	2019	100,283	72,083	61,507
		2020	98,640	56,315	58,665
		*p*	0.409	< 0.001*	0.387
		% change	−1.6%	−21.9%	−4.6%
	Male	2019	41,398	29,667	25,546
		2020	40,424	23,278	24,421
		*p*	0.285	< 0.001*	0.410
		% change	−2.4%	−21.6%	−4.4%
	Female	2019	58,885	42,416	35,961
		2020	58,216	33,037	34,244
		*p*	0.570	< 0.001*	0.374
		% change	−1.1%	−22.1%	−4.8%
Elderly	Overall	2019	60,830	45,153	38,077
		2020	62,555	43,307	39,222
		*p*	0.181	0.333	0.517
		% change	+2.8%	−4.1%	+3.0%
	Male	2019	24,904	18,241	15,296
		2020	25,545	17,656	16,061
		*p*	0.245	0.411	0.291
		% change	+2.6%	−3.2%	+5.0%
	Female	2019	35,926	26,912	22,781
		2020	37,010	25,651	23,161
		*p*	0.160	0.306	0.726
		% change	+3.0%	−4.7%	+1.7%

*Note:* Significant differences between 2019 and 2020 (*p* < 0.05) are indicated by ‘*’. In addition, the percentual (%) change from 2019 to 2020 was reported.

In the first half of 2020, the total number of first‐time dispensed analgesics (*n* = 367,094) was significantly lower than in the same period of 2019 (*n* = 388,973; *p* = 0.021). This difference was particularly notable during the COVID‐19 lockdown period (weeks 12–19), where the number of new analgesic users in 2020 was significantly lower compared to 2019 (*p* = 0.003). No significant differences were observed for the 2 years for the pre‐lockdown (weeks 1–11) and post‐lockdown (weeks 20–26) periods.

When comparing the three periods within each year, a significant decline in first‐time analgesic dispensing was observed across all intervals in 2019 (*p* = 0.001). Specifically, compared to weeks 1–11, the number of first‐time dispensations significantly decreased during weeks 12–19 (*p* < 0.001) and weeks 20–26 (*p* < 0.001), and a further reduction was observed from weeks 12–19 to weeks 20–26 (*p* < 0.001). A similar pattern was observed in 2020, with significant reductions in newly dispensed analgesics during both weeks 12–19 (*p* < 0.001) and weeks 20–26 (*p* < 0.001), compared to weeks 1–11. However, in contrast to 2019, no significant difference was found between the pre‐lockdown (weeks 1–11) and post‐lockdown (weeks 20–26) periods in 2020 (*p* = 0.705) (see Figure 1).

Newly prescribed analgesics were most frequently dispensed to adults, followed by the elderly, adolescents and children. In both 2019 and 2020, analgesics were significantly more often dispensed to females than males (*p* < 0.001), with the exception of children, for whom no overall sex differences were observed (see Table 1).

In the pre‐lockdown period (weeks 1–11), no significant differences were found in the number of first‐time dispensed analgesics for children, adults, or the elderly when comparing 2020 and 2019. However, among adolescents (overall and females), a significant reduction of 6.6% in first‐time dispensations was observed in 2020. No significant difference was found for adolescent males during this period.

**FIGURE 2:**
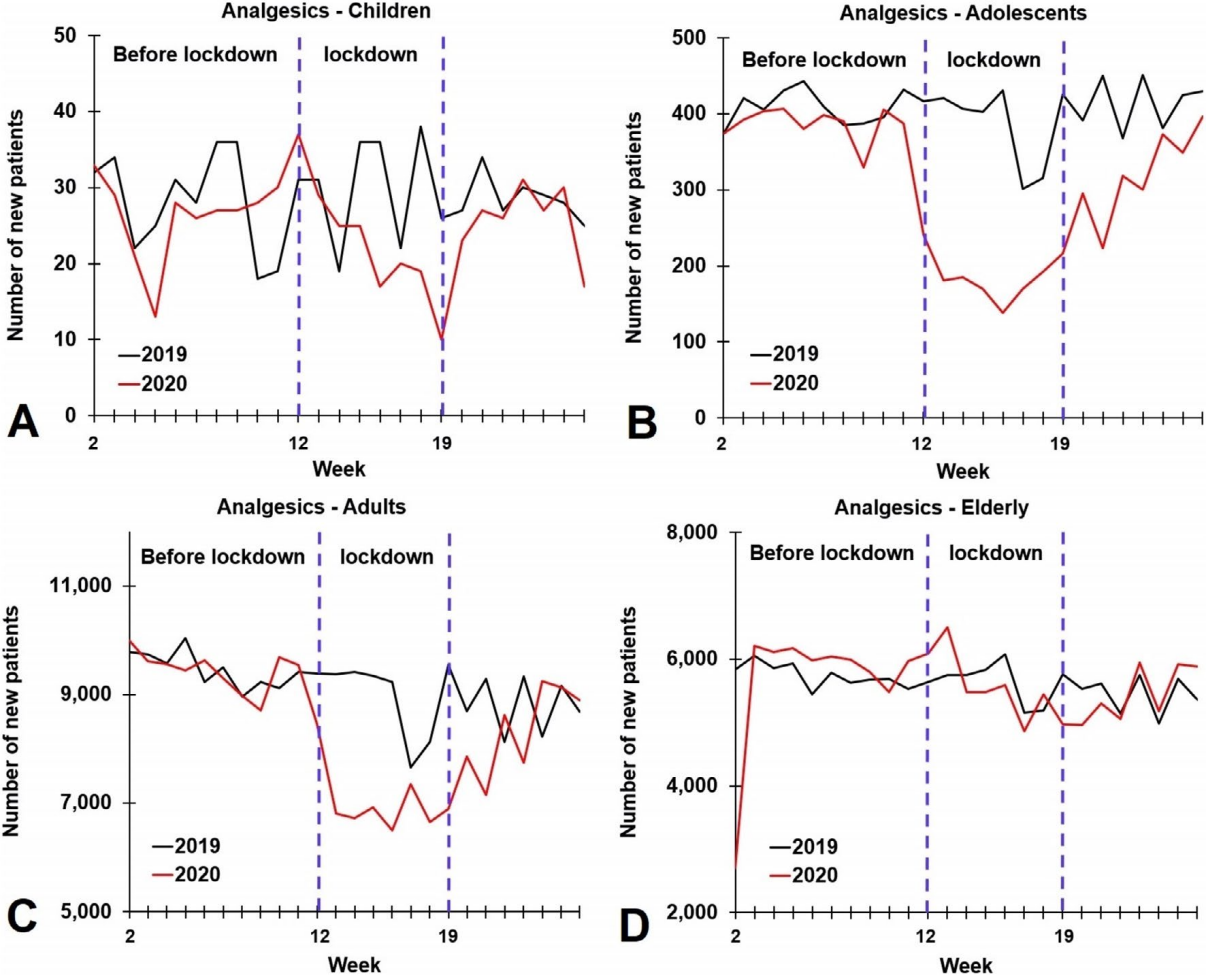
Number of newly dispensed analgesics in 2019 and 2020 according to age group. Data are shown according to age group: (A) children, (B) adolescents, (C) adults and (D) the elderly.

During the lockdown period (weeks 12–19), a significant reduction in first‐time dispensed analgesics was observed in 2020 compared to 2019 among both adolescents and adults (see Figure 2). These reductions were significant for both males and females in these age groups (*p* < 0.01 for all comparisons). Although a notable decline in first‐time dispensations was found for children (−23.8%), the difference did not reach statistical significance. No significant differences were observed for the elderly (see Table 1). In the post‐lockdown period (weeks 20–29), no significant year‐to‐year differences were observed for children, adults, or the elderly. However, among adolescents (overall and males), a significant decline in first‐time dispensed analgesic drugs was noted in 2020 compared to 2019 (both *p* < 0.01).

## 4 Discussion

A significant reduction in first‐time dispensations of analgesics was observed among adolescents and adults during the first COVID‐19 lockdown period in 2020. Also, in children, a relevant drop in first‐time dispensed analgesics was noted during the first lockdown period (−23.8% compared to 2019), but due to the relatively small sample size, the difference was not statistically significant. No significant differences between 2019 and 2020 were found for the elderly.

As pharmacies in the Netherlands remained open during this time, the observed decline is likely reflective of broader delays in accessing healthcare, rather than limited availability of medications. These findings align with previous research on the impact of the COVID‐19 pandemic on healthcare in the Netherlands (Nab et al. 2021; RIVM 2021; De Graaff et al. 2022; Mizee et al. 2022).

One study reported that approximately 20% of emergency department visitors experience delays in seeking care, with nearly half attributing these delays to the pandemic (Nab et al. 2021). Notably, 16.5% of respondents believed their symptoms would have been less severe, and 9.4% believed their conditions might have been preventable had they received care earlier (Nab et al. 2021). Similar patterns were seen in surgical care, with a marked decrease in procedures during the pandemic (De Graaff et al. 2022). Postponed care‐seeking behaviour was also reported among older adults, although the prevalence of cancelled appointments declined from 35% in 2020 to 17% in 2021. However, the rate of delayed help‐seeking remained consistent across both years (Mizee et al. 2022). No significant differences in analgesic prescription rates were observed for children or the elderly between 2019 and 2020. One explanation may be that adolescents and adults faced greater disruptions to their routines due to responsibilities such as school, work, or caregiving, potentially discouraging them from seeking medical attention (RIVM 2021). In contrast, older adults may have received prioritised care during this period. This is supported by data showing an increase in the average age of surgical patients in 2020 compared to 2018 and 2019, suggesting a shift in prioritisation toward older individuals (De Graaff et al. 2022). Of note, first‐time prescriptions in the Netherlands usually require a physical visit to a physician. However, during the pandemic there was an increase in telehealth replacing physical visits (Bos et al. 2020; Van Tuyl et al. 2020). This may have prevented a further decline in first‐time prescriptions during the pandemic, in particular during the lockdown periods. Further, pharmacies also delivered medication by post. Increased home delivery of medicines may have reduced the impact of avoiding pharmacies as a driver for the observed reduced first‐time dispensed analgesic drugs.

There are restrictions and strict regulations in the Netherlands regarding the delivery of analgesic drugs that are listed under the opiate law (KNMP 2025; Wettenbank Overheid.nl 2025). However, in contrast to other countries, these regulations were not changed during the COVID‐19 pandemic. As such, a difference in ease of access of analgesics cannot explain the current findings. However, in the Netherlands, during the COVID‐19 pandemic there was a shift from in‐person to digital physician consults (RIVM 2021). During the pandemic, physicians received the same payment for in‐person and digital consults. Also, the number of home deliveries of medicines increased (RIVM 2021). These changes did not result in an increase in prescriptions and first‐time dispensations. Instead, the current data showed a reduction of first‐time analgesic drug dispensations during the first lockdown period.

A key strength of this study is its focus on newly dispensed analgesics, which allows for a more precise analysis of changes in prescription behaviour. The stratification by age and sex further strengthens the study by enabling more accurate detection of group‐specific effects. However, this study also has some limitations. For example, it remains unclear to what extent individuals may have turned to OTC painkillers instead of prescription medications to manage their pain. Due to fear of SARS‐CoV‐2 infection, individuals may have avoided visiting doctors and pharmacies. Alternatively, they may have self‐medicated with OTC painkillers, which were home‐delivered or available from supermarkets. Also, sales data revealed that at the beginning of the COVID‐19 pandemic, people were stockpiling OTC medication, including analgesics (Ray et al. 2022). However, it is unknown whether these medications have been actually used. Additionally, it is further unknown how much of the observed decline can be attributed to the reduction in surgery during the lockdown. With fewer people working on location, reduced commuting, and the closure of sports venues, there may have been a decline in travel‐, sports‐, and work‐related accidents and injuries—factors that could have contributed to a lower demand for analgesics. Finally, the current analyses were limited to the first half year of the COVID‐19 pandemic and the effects of the first lockdown period. During the pandemic, several subsequent lockdown periods were instituted. It would be interesting to further analyse the impact of subsequent lockdown periods on the dispensation of analgesic drugs. In these studies, information could also be gathered about the specific types of analgesics that were dispensed, and what type of pain was treated.

In conclusion, the data indicate a reduction in first‐time analgesics prescriptions, particularly among adolescents and adults. This suggests that a substantial number of patients may have experienced untreated pain during the first Dutch COVID‐19 lockdown. Future research should explore the consequences of lower prescription rates during such disruptions, how patients managed their pain in the absence of prescriptions, and the extent to which OTC alternatives were used. Together, these findings highlight the need to evaluate and strengthen pain management policies in preparation for future healthcare disruptions.

## Author Contributions

Conceptualization: M.N.Z., P.K., P.A.H., D.M.D., J.G. and J.C.V. Data curation and formal analysis: J.C.V. Writing – original draft preparation: M.N.Z. and J.C.V. Writing – review and editing: M.N.Z., P.K., P.A.H., D.M.D., J.G. and J.C.V. All authors have read and agreed to the published version of the manuscript.

## Conflicts of Interest

The authors have no conflicts of interest to declare in relation to the current work. Over the past 36 months, J.C.V. received research grants from Danone and Inbiose and has acted as a consultant/expert advisor to Eisai, KNMP, Med Solutions, Mozand, Red Bull, Sen‐Jam Pharmaceutical and Toast! J.C.V. has received travel support from Sen‐Jam Pharmaceutical and owns stock in Sen‐Jam Pharmaceutical. J.G. is a part‐time employee of Nutricia Research and received research grants from Nutricia Research Foundation, Top Institute Pharma, Top Institute Food and Nutrition, GSK, STW, NWO, Friesland Campina, CCC, Raak‐Pro and EU. M.N.Z. and D.M.D. have received travel support from Sen‐Jam Pharmaceutical. P.K. is CEO of Pangenix. P.A.H. has nothing to declare.
